# Case Report: Spontaneous regression of extruded lumbar disc herniation following Chuzhen therapy over 3 years

**DOI:** 10.3389/fmed.2025.1674464

**Published:** 2025-10-29

**Authors:** Youpeng Hu, Ye-Hui Wang, Wenlong Guo, Shengxin Zhao, Peidong Qing, Shiming Xie, Xiaohong Fan

**Affiliations:** ^1^Spine Department, Mianyang Orthopaedic Hospital, Mianyang, China; ^2^Spine Department, Sichuan Province Orthopedic Hospital, Chengdu, China; ^3^Spine Department, Hospital of Chengdu University of Traditional Chinese Medicine, Chengdu, China

**Keywords:** Chuzhen therapy, lumbar disc herniation, spontaneous regression, pestle needle, protruding nucleus pulposus

## Abstract

**Background:**

Lumbar disc herniation (LDH) is a common disease, and most cases can be satisfactorily treated with conservative therapy. Chuzhen therapy, a non-surgical, non-pharmacological, and non-invasive therapy, can be performed at home, making it more acceptable to patients. However, evidence supporting the efficacy of this therapy for the treatment of acute LDH is limited.

**Case presentation:**

We report a 50-year-old male who presented with lower back pain, persistent radiating pain, and numbness in the lower extremities. Computed tomography (CT) imaging revealed a large right-sided protruding nucleus pulposus (PNP) at the L5/S1 level, compressing the nerve root. The patient experienced significant symptomatic relief after 1 month of outpatient Chuzhen therapy followed by 3 years of home-based Chuzhen therapy. Follow-up imaging conducted 3 years later showed a remarkable regression of the herniated nucleus pulposus, with a substantial 64.8% reduction in its size. Concurrently, the patient's LDH classification was downgraded from MSU3-AB to MSU2-A.

**Conclusions:**

We report for the first time a case of LDH treated at home with Chuzhen therapy, which resulted in disc retraction. In this case, Chuzhen therapy was safe and effective as a monotherapy, but more conclusive and effective evidence is needed to elucidate its specific therapeutic mechanism.

## Background

**Figure 1:**
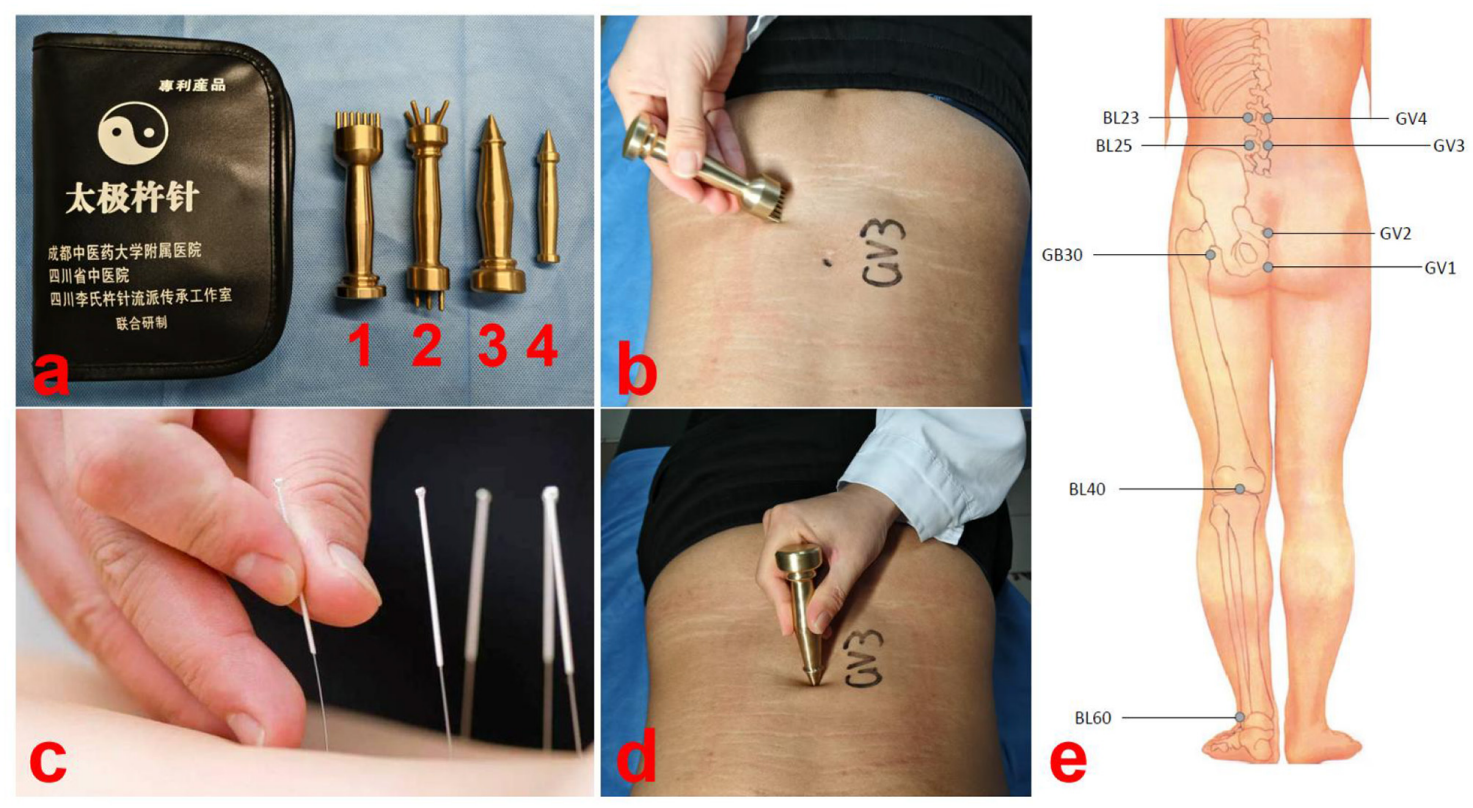
Demonstration diagram of Chuzhen therapy. **(a)** Chuzhen therapy involves the use of four blunt-ended needles made of brass (No. 1: *Qiyao Hunyuan* Chuzhen, No. 2: *Wuxing Santai* Chuzhen, No. 3: *Jingang* Chuzhen, and No. 4: *Kuixing* Chuzhen). **(b)** Using the tip of No. 1 Chuzhen, we performed the Tai Chi technique seven times in a counterclockwise direction at the center of the Yaoyangguan acupoint (GV3). **(c)** Demonstration diagram of acupuncture. Acupuncture requires piercing the skin, while Chuzhen does not. **(d)** Performing 49 taps with the No. 2 Chuzhen at the acupoint yaoyangguan (GV3). **(e)** Locations of Chuzhen therapy.

Lumbar disc herniation (LDH) is a condition in which the nucleus pulposus protrudes through a ruptured annulus fibrous, compressing or irritating the adjacent nerve roots and paravertebral tissues, resulting in symptoms such as back pain or numbness in the lower extremities. The treatment of LDH is divided into surgical and non-surgical approaches. Non-surgical treatments for LDH are gaining attention and preference, as most patients are apprehensive of undergoing surgery (1). Several studies have shown no significant difference in medium or long-term outcome between surgical and non-surgical treatments for LDH (2–6). Acupuncture is a well-known and effective non-surgical treatment (7–9) (Figure 1c). Other manual therapies have also shown effectives on restoration of disc herniation (10, 11). Chuzhen therapy is a unique traditional Chinese medicine therapy with proven efficacy, especially in the treatment of orthopedic lower back pain, and has been included in the National Intangible Cultural Heritage Project (12–14). This non-pharmacological, non-invasive therapy is simple to operate and easy to learn, enabling rapid training of healthcare providers and home-based administration by patients. Chuzhen therapy has gained international recognition and has been adopted in numerous countries. In this article, we report the case of a patient with LDH who had significantly reduced the size of the PNP and improved pain after 3 years of Chuzhen treatment at home.

## Case presentation

### Patient information

The patient was a 50-year-old male driver who presented to our hospital on August 29, 2021, with unexplained lower back pain persisting for hours or days. Over the preceding 2 months, his lower back pain aggravated after prolonged sitting and was relieved with hot compression or rest, which led him to initially dismiss the symptoms. Two days before the current consultation, the pain intensified following a sprain and developed radiating pain and numbness along the posterior side of right lower limb, which seriously affected his life and sleep. His maximum walking distance was approximately 100 m. He had no other relevant medical history and was not taking any medication or receiving any treatment. The patient was 165 cm tall, weighed 64 kg, and had a body mass index of 23.4 kg/m^2^.

### Diagnostic assessment

Physical examination revealed significant percussion and pressure pain in the spinous processes and bilateral paraspinal muscles at the L5/S1 level. Radiating pain, involving the lower extremities, extended from the right buttock to the posterior of the right calf. A Lasseger test on the right leg was positive at 25°, and the test for abdominal external pressure was also positive. The strength of the right extensor hallucis longus and extensor digitorum muscles was graded 4, and hypoesthesia was observed on the posterior aspect of the right calf and lateral ankle. The muscle strength of the remaining muscle groups was normal, with no ankle clonus present, and no pathological reflexes were elicited in either lower extremity. The patient's lower back and radicular pain intensities were measured at 8 points on a 10-point visual analog scale (VAS), and the Oswestry Disability Index (ODI) and lumbar Japanese Orthopedic Association (JOA) scores were 45 and 7, respectively.

**Figure 2:**
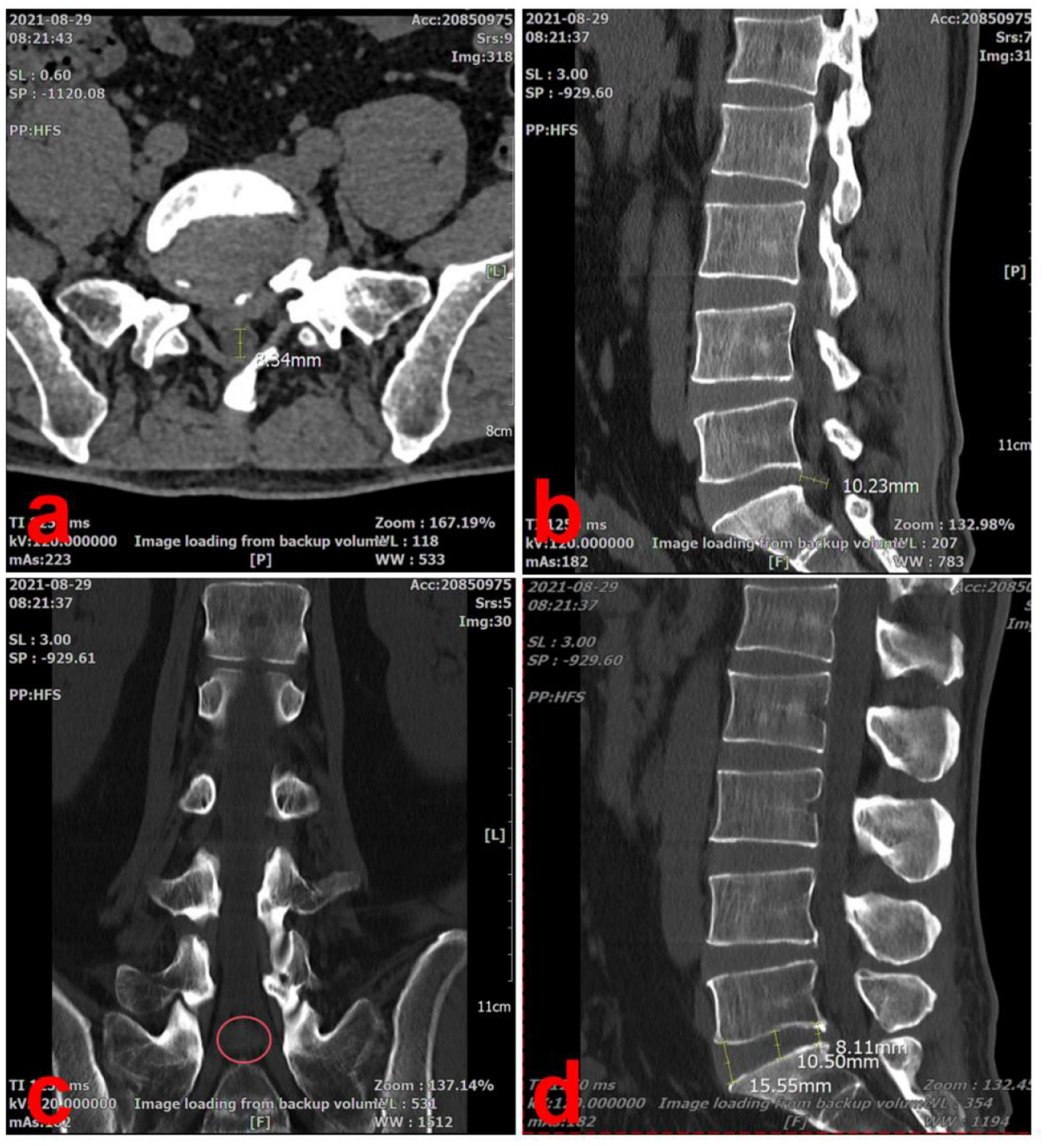
Lumbar spine CT scan at the initial visit (2021-08-29). **(a)** On the cross-section view, the diameter of the spinal canal at the L5/S1 level was 8.34 mm. **(b)** On the sagittal view of the L5/S1 intervertebral space, the herniated disc measured 10.23 mm in length. **(c)** The coronal view, the herniated disc was compressing the right S1 nerve root, consistent with the patient's symptoms (red circle). **(d)** On the sagittal view, the height of the lumbar intervertebral space was 11.83 mm.

A computed tomography (CT) scan showed a large right-sided disc herniation at the L5/S1 level, with an Michigan State University (MSU) classification of 3-AB (15) (Figure 2a). On the sagittal view of the L5/S1 intervertebral space, the herniated disc measured 10.23 mm in length and the height of the lumbar intervertebral space was 11.83 mm (Dabbs method: the height of the front + posterior edge/2) (Figures 2b, d). On the cross-section view, the diameter of the spinal canal at the L5/S1 level was 8.34 mm (Figure 2a). The coronal view, the herniated disc was compressing the right S1 nerve root, consistent with the patient's symptoms (Figure 2c).

By combining physical examination and imaging findings, we were able to definitively diagnose the patient with LDH (L5/S1, MSU: 3-AB) and secondary lumbar spinal stenosis.

### Therapeutic intervention

Percutaneous endoscopic lumbar discectomy was recommended; however, the patient refused surgery and opted for conservative treatment. Simultaneously, as the patient refused oral medications and invasive treatments, choosing instead to undergo Chuzhen therapy as conservative treatment. Chuzhen therapy involves the use of four blunt-ended needles made of brass (No. 1: *Qiyao Hunyuan* Chuzhen, No. 2: *Wuxing Santai* Chuzhen, No. 3: *Jingang* Chuzhen, and No. 4: *Kuixing* Chuzhen), ~10 cm long (Figure 1a).

We developed a Chuzhen therapy plan based on his symptoms and diagnosis, and the patient underwent 20 sessions (5 sessions per week for 4 weeks). The acupuncture points were Changqiang (GV1), Yaoshu (GV2), Yaoyangguan (GV3), Mingmen (GV4), Huantiao (GB30), Shenshu (BL23), Dachangshu (BL25), Weizhong (BL40) and Kunlun (BL60) (Supplementary Table 1 and Figure 1e). First, using the tip of No. 1 Chuzhen, we performed the Tai Chi technique seven times in a counterclockwise direction at the center of the Yaoyangguan acupoint (GV3) (Figure 1b). Second, 49 taps were performed using Chuzhen No. 2 at the Yaoyangguan acupoint (GV3) (Figure 1d). Third, along the Hechelu line from Changqiang (GV1) to Mingmen (GV4) and six adjacent lines, the tip of No. 1 Chuzhen was moved from top to bottom seven times per lines, for a total of 49 times. Finally, using the tip of No. 3 Chuzhen and D, 49 taps were performed at the Huantiao (GB30), Shenshu (BL23), Dachangshu (BL25), Weizhong (BL40), and Kunlun (BL60) acupoints. Each session lasted ~20 min. The Treatment intensity refers to the level at which skin flushing is induced while remaining within the patient's tolerance range. No bruising or other adverse effects were observed.

### Follow-up and outcomes

**Figure 3:**
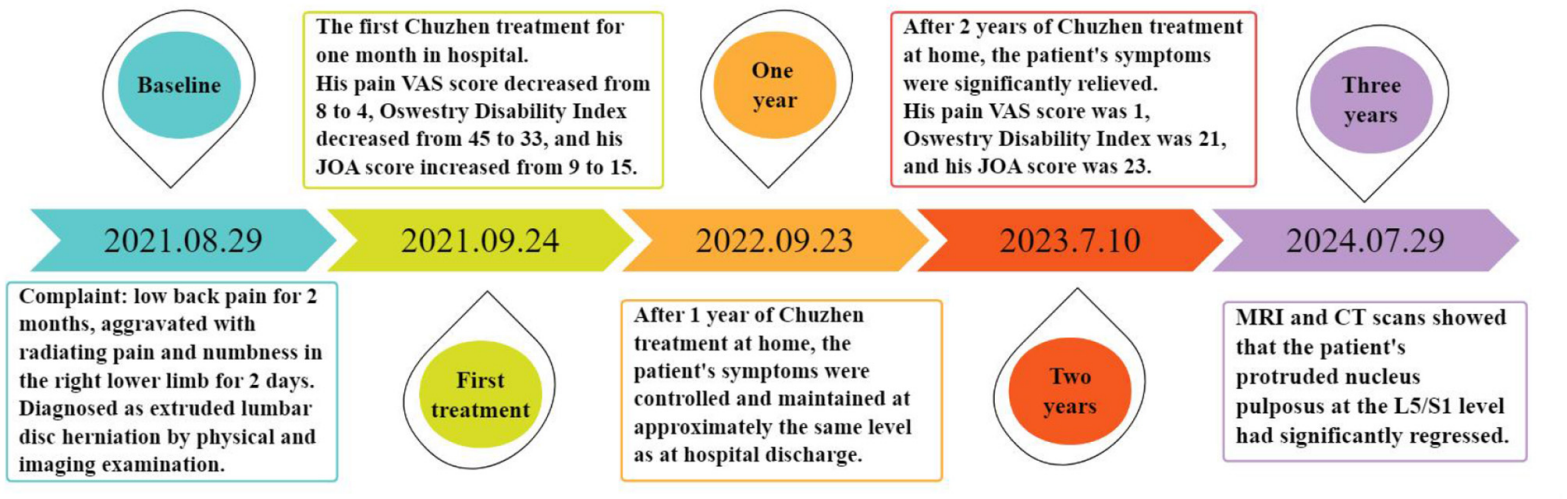
The timeline of the patient's treatment.

After 1 month of Chuzhen treatment, the patient's pain intensity decreased from 8 to 4 on the VAS, lumbar JOA score improved from 9 to 15, and ODI score decreased from 45 to 33. However, due to financial obligation requiring him to work as a driver, the patient was unable to continue visiting the hospital for Chuzhen treatment. During the treatment period, his wife, who is a nurse, learned about Chuzhen therapy as an easy-to-handle and non-invasive treatment technique. Therefore, she enrolled in a 2-week training course on Chuzhen therapy organized by us on a regular basis and received a certificate of competency. His wife provided Chuzhen treatment at home to prevent the recurrence of symptoms, enabling the patient to work long hours off-site as a driver without worrying. The treatment regimen administered at home is identical to that provided in hospital settings, with the patient's spouse responsible for monitoring and documenting progress. Adherence is further ensured through online check-ins and quarterly follow-up appointments. The timeline of the patient's treatment is shown in Figure 3. Symptoms initially recurred at the end of the outpatient treatment but were significantly relieved after 1 year and nearly eliminated after 3 years. During follow-up period nearly 3 years, the patient had no complications. The evolution of the patients' VAS, JOA and ODI scores over the course of treatment is summarized in Supplementary Figure 1.

**Figure 4:**
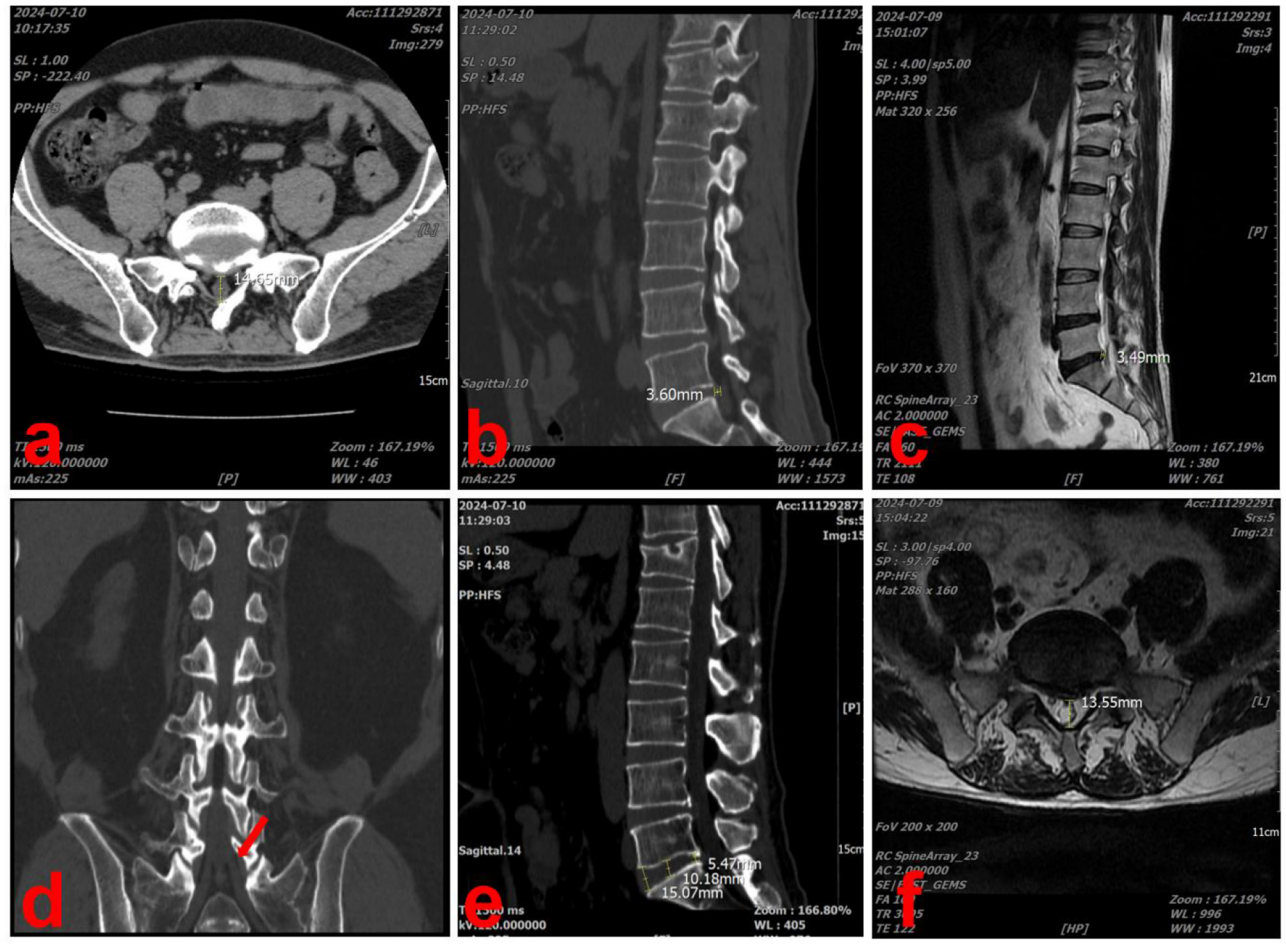
Lumbar spine CT and MRI scans performed at the last visit (2024-07-09). **(a)** In the CT cross-section, the diameter of the spinal canal at the L5/S1 level was 14.65 mm. **(b)** In the CT sagittal-section, the herniated disc measured 3.60 mm in length. **(c)** In the MRI sagittal-section, the herniated disc measured 3.49 mm in length. **(d)** In the coronal section, there was no longer compression of the S1 nerve root by the herniated disc (red arrow). **(e)** In the CT sagittal-section, the height of the lumbar intervertebral space was 10.27 mm. **(f)** In the MRI cross-section, the diameter of the spinal canal at the L5/S1 level was 13.55 mm.

At the most recent follow-up, on July 9, 2024, the patient underwent CT and magnetic resonance imaging (MRI) of the lumbar spine, which revealed significant resorption of the herniated disc. In the sagittal section of the L5/S1 intervertebral space, the herniated disc measured 3.60 mm in length and the height of the lumbar intervertebral space was 10.27 mm (Figures 4b, e). In the cross-section, the diameter of the spinal canal at the L5/S1 level was 14.65 mm (Figure 4a). In the coronal section, there was no longer compression of the S1 nerve root by the herniated disc (Figure 4d). The results of MRI were similar to those of CT, though minor discrepancies may have arisen due to measurement and positional factors (Figures 4c, f). The patient's LDH classification was downgraded from MSU3-AB to MSU2-A and secondary lumbar spinal stenosis was alleviated.

## Discussion and conclusions

Symptomatic degenerative disc disease is estimated to affect approximately 5.5% of the global population, with a lifetime risk of LDH estimated at 30% (16, 17). While some patients with herniated lumbar discs heal conservatively or spontaneously, others require surgery. Conditions that generally require surgery include a large protruding nucleus pulposus, especially when the PNP breaks through the posterior longitudinal ligament and enters the subdural space or when the PNP completely breaks away from the annulus fibrosis and falls into the subdural space (18). However, many patients with extruded or sequestered discs refuse surgical treatment, and approximately 60%−90% of LDH cases can be successfully treated using a conservative approach alone (19, 20). Spontaneous regression of PNP can be completely resolved with conservative treatment (21–23). The probability of spontaneous regression was significantly higher in extruded and sequestered discs than that for discs with a smaller morphology (24).

Since the first report of LDH regression in 1984 by Guinto et al. (25), an increasing number of studies have characterized spontaneous regression of LDH using CT and MRI (26–28). With the growing number of case reports regarding LDH regression, a number of scholars have proposed the hypothesis that there is an inevitable link between PNP regression and the disappearance of clinical symptoms and that the regression or disappearance of PNP is the main reason for the efficacy of conservative treatments in patients with LDH (29–31). For example, Demirel et al. (32) designed a double-blind randomized controlled trial to confirm that non-invasive spinal decompression therapy is effective in improving pain, resorbing PNP, and increasing disc height.

There are three hypotheses regarding the mechanism of the spontaneous PNP regression: (1) Inflammatory infiltration and neovascularization. Herniated discs can stimulate autoimmune inflammatory responses and neovascularization to induce phagocytosis, resulting in PNP regression. This mechanism has been recognized by many researchers (33–40). Ikeda et al. (41) revealed inflammatory cell infiltration, granulation, and neovascularization in 100% of extruded discs and in 80% of sequestrated discs using histology and immunohistochemistry. Iwabuchi et al. (42) found that a PNP readily induces immunophagocytosis, whereas a protruding annulus fibrosis does not, demonstrating that the development of immune inflammation is dependent on the blood supply. (2) Nucleus pulposus dehydration. During the acute phase of LDH, the PNP becomes locally edematous, followed by gradual dehydration and retraction into the annulus fibrosus (43). Henmi et al. (44) observed an increase in MRI signal intensity ratio after the acute phase of LDH, suggesting that breakthrough of the annulus fibrosis by the PNP may cause the formation of localized edema. Yang et al. (45) found that the PNP retracts into the intervertebral space when it passes through the annulus fibrosis without separating from it, suggesting that dehydration plays a critical role in spontaneous PNP absorption, but this mechanism does not explain the complete disappearance of PNP. (3) Enzymatic degradation and phagocytosis. PNP retraction is associated with an imbalance in the matrix-degrading enzyme system, such as matrix metalloproteinase-3 (MMP-3) and tissue inhibitor of metalloprotein-1 (46, 47). Meng found that thrombin G and L may upregulate MMP-1 and MMP-3, facilitating the degradation of the extracellular matrix and spontaneous resorption of disc herniation (48). To date, no literature has reported definitive mechanisms by which Chuzhen therapy or other conservative treatments induce disc retraction. Consequently, we can only speculate that Chuzhen therapy may stimulate the fascia to accelerate blood circulation and promote neovascularisation, thereby enhancing the autoimmune inflammatory responses to induce phagocytosis.

The review of the literature revealed that the time frame for PNP retraction is generally no less than 2 months, and that for complete resorption is no < 6 months, regardless of whether conservative treatment or rest alone is received (27, 49–51). After PNP retraction, nerve root compression and irritation are reduced, which can positively affect patient recovery from spinal mechanical imbalance. In addition, there is a positive psychological impact when patients learn about PNP retraction, which helps them overcome anxiety associated with long-term illness. However, some researchers believe that the remission of clinical symptoms is not significantly associated with PNP retraction (52–54). Numerous case reports have documented spontaneous resorption of herniated discs with conservative therapy; however, most studies have been limited by a short follow-up period.

We report this case because the patient's occupation as a driver, which requires prolonged sedentary periods, may be a risk factor for the onset of LDH. Given the patient's refusal to undergo surgery, oral medications, or invasive treatments, we used Chuzhen therapy as a conservative treatment. Chuzhen therapy is a long-standing treatment that has persisted for >300 years and is an important branch of external treatment in traditional Chinese medicine that uses a set of specialized tools which are pestle-shaped non-invasive meridian and acupoint stimulators, also known as “pestle needles”. This therapy combines the advantages of acupuncture and massage, but the “needle” does not penetrate the skin, making it a noninvasive and drug-free therapy. Chuzhen therapy dredges the meridians and collaterals, relieves fatigue, reduces muscle and fascial tension, and locally alleviates sterile inflammation (12–14, 55, 56). This therapy has been used in several countries including Germany, the Netherlands, France, and the United Kingdom. Regular training courses have enabled thousands of Chinese and foreign students, including doctors and nurses, to become proficient in Chuzhen therapy, typically after 2 weeks of training.

The patient received Chuzhen treatment for 1 month at the hospital and 3 years at home. The patient's symptoms were significantly relieved after 3 years of follow-up. His VAS pain score decreased from 8 to 0, ODI decreased from 45 to 5, and the JOA score increased from 7 to 24. Moreover, imaging findings also demonstrated notable improvements, including a 64.8% shortening of the sagittal herniated length, 19.2% enlargement of the spinal canal diameter, and no significant changes in intervertebral height. This case highlights the long-term efficacy of Chuzhen therapy for LDH and suggests that its therapeutic mechanism may be related to the spontaneous resorption of disc herniation observed during the three-year follow-up. The 3-year regression timeline aligns with faster-resorbing cohorts, possibly enhanced by Chuzhen therapy's proposed neovascularization effects. However, without controls, we cannot exclude natural history.

This study had certain limitations: (1) The time points of the imaging observations were only at the first and last follow-up visits, which did not clarify the exact time and rate of spontaneous resorption of disc herniation. Perhaps conducting imaging examinations at the point when the patient's symptoms show marked improvement during the first year could provide a more compelling explanation. (2) The spontaneous resorption of the patient's intervertebral disc protrusion may stem from self-healing or a placebo effect. Current evidence does not establish a causal relationship between Chuzhen therapy and PNP. (3) This paper is just a single case report, with a solitary sample and no control group. Through this interesting case, we proposed the conjecture that the mechanism of Chuzhen therapy for LDH may be related to the retraction of the PNP. Large-scale randomized controlled trials are needed to validate these findings.

This report presents Chuzhen therapy as a non-pharmacological, non-invasive, and easy-to-learn treatment that can be administered at home. The mechanism of Chuzhen therapy for LDH may be related to the spontaneous resorption of the herniated disc. Such therapy provides a conservative treatment option for certain patients with LDH who refuse surgical treatment. While Chuzhen therapy coincided with disc regression, controlled trials are needed to establish efficacy beyond natural history. Home-based administration shows promise for compliant patients.

## Data Availability

The original contributions presented in the study are included in the article/Supplementary material, further inquiries can be directed to the corresponding authors.
