# Supplementary material for: Case Report: Spontaneous regression of extruded lumbar disc herniation following Chuzhen therapy over 3 years
Source: Front Med (Lausanne). 2025 Oct 29;12:1674464. doi: 10.3389/fmed.2025.1674464 (PMC12605046; doi:10.3389/fmed.2025.1674464)

**Supplementary Table and Fig**

Table. 1 Locations of Chuzhen therapy

| Acupoints | Locations |
| --- | --- |
| Changqiang (GV1) | In the perineal region, below the tip of the coccyx, at the midpoint between the tip of the coccyx and anus. |
| Yaoshu (GV2) | On the sacrum, on the posterior midline in the hiatus of the sacrum. |
| Yaoyangguan (GV3) | In the lumbar region, on the posterior midline, in the depression below the spinous process of the 4th lumbar vertebra. |
| Mingmen (GV4) | In the lumbar region, on the posterior midline, in the depression below the spinous process of the 2rd lumbar vertebra. |
| Huantiao (GB30) | On the gluteal region, at the junction of the middle 2/3 and lateral 1/3 of the line joining the prominence of the great trochanter and the sacral hiatus. |
| Weizhong (BL40) | On the posterior region of knee, at the midpoint of the transverse crease of the popliteal fossa, between the tendons of the biceps of the thigh and the semitendinous . |
| Kunlun (BL60) | On the ankle region, in the depression between the lateral malleolus and the Achilles's tendon. |
| Shenshu (BL23) | In the lumbar region,1.5 cun lateral to the depression below the spinous process of the 2nd lumbar vertebra. |
| Dachangshu (BL25) | In the lumbar region,1.5 cun lateral to the depression below the spinous process of the 4th lumbar vertebra. |

Supplementary Figure. 1 The evolution of the patients’ VAS, JOA and ODI scores over the course of treatment.


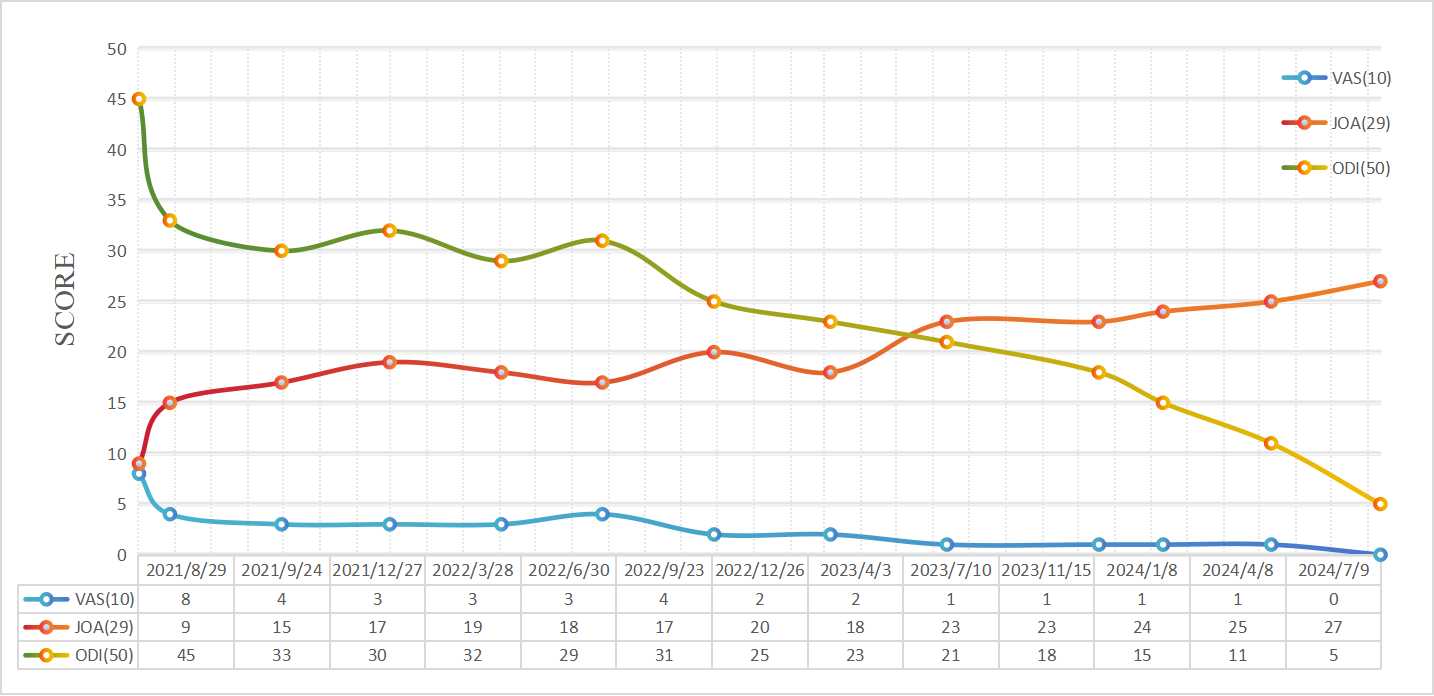

Supplement: Supplementary file 1 [file Data_Sheet_1.docx]
